# Absence of Crimean-Congo haemorrhagic fever virus in the tick *Hyalomma aegyptium* parasitizing the spur-thighed tortoise (*Testudo graeca*) in Tunisia

**DOI:** 10.1051/parasite/2019036

**Published:** 2019-06-14

**Authors:** Wasfi Fares, Khalil Dachraoui, Chawki Najjar, Hend Younsi, Stephen Findlay-Wilson, Marie Petretto, Stuart Dowall, Roger Hewson, Elyes Zhioua

**Affiliations:** 1 Institut Pasteur de Tunis, Laboratory of Vector Ecology 13 Place Pasteur 1002 Tunis Tunisia; 2 Marwell Wildlife Colden Common Thompsons Lane Winchester SO21 1JH Hampshire UK; 3 Institut Supérieur des Sciences Biologiques Appliquées de Tunis, Université Tunis El Manar 9 Avenue Zouhaïer Essafi 1009 Tunis Tunisia; 4 Public Health England Manor Farm Road, Porton Down Salisbury SP4 0JG Wiltshire UK

**Keywords:** Crimean-Congo haemorrhagic fever virus, *Hyalomma aegyptium*, *Testudo graeca*, North Africa

## Abstract

Free-ranging spur-thighed tortoises *Testudo graeca*, captured in different habitat types of Northern Tunisia from March to April 2017, were examined for tick infestation: 134/147 (91%) were infested. The overall infestation intensity and abundance was 8.5 and 7.8, respectively. From these tortoises, 1174 ticks were collected, of which 10% (*n* = 120) taken from 18 randomly-selected tortoises were identified at the species level; the remaining ticks were examined for the presence of Crimean-Congo haemorrhagic fever virus (CCHFv) by real time RT-PCR. Only adult *Hyalomma aegyptium* were found, suggesting a high degree of host specificity to tortoises. No CCHFv was detected in ticks. Considering the absence of CCHFv in *Hyalomma aegyptium* infesting its main host, the spur-thighed tortoise, this tick species is unlikely to play a major role in the epidemiology of CCHF. Therefore, more studies are needed to investigate the circulation of this arbovirus between livestock and other tick species from North Africa.

## Abstract (fr)

Des tortues grecques, *Testudo graeca*, capturées dans différents types d’habitats du nord de la Tunisie de mars à avril 2017 ont été examinées pour la présence de tiques : 134/147 (91 %) étaient infestées. L’intensité d’infestation et l’abondance globale étaient respectivement de 8,5 et 7,8. Des tiques (n = 1174) ont été collectées de ces tortues, dont 10 % (n = 120) prélevées sur 18 tortues sélectionnées au hasard ont été identifiées au niveau de l’espèce, et les tiques restantes ont été examinées pour la présence du virus de la fièvre hémorragique de Crimée-Congo (vFHCC) par RT-PCR. Seuls des *Hyalomma aegyptium* adultes ont été trouvés, ce qui suggère un degré élevé de spécificité de cette espèce de tique pour les tortues Aucun vFHCC n’a été détecté dans les tiques. Compte tenu de l’absence du vFHCC chez *Hyalomma aegyptium* collecté de tortues (*Testudo graeca*), cette espèce de tique ne joue probablement pas un rôle important dans l’épidémiologie de la FHCC. Par conséquent, d’autres études sont nécessaires pour investiguer la circulation de cet arbovirus entre le bétail et d’autres espèces de tiques d’Afrique du Nord.

## Introduction

Crimean-Congo haemorrhagic fever (CCHF), a tick-borne disease caused by the Crimean-Congo haemorrhagic fever virus (CCHFv) (family Bunyaviridae, genus *Nairovirus*) is characterized by fever and hemorrhage with case fatality rates of 9–50% [10, 43]. CCHFv is transmitted in nature mainly by hard ticks of the genus *Hyalomma* [13, 36]. Ticks are considered a vector and also a reservoir because they are able to maintain the virus during its life cycle by transstadial transmission and can be passed onto offspring by transovarial transmission [47]. CCHFv is maintained in nature through a transmission cycle involving ticks and vertebrate hosts, mainly domestic animals such as cattle, and sheep [21]. CCHF has been reported in Africa [29, 44], Asia [28], the Middle East [34], and Eastern Europe [17, 46]. In the Western Mediterranean Basin, the first case of CCHF was reported from Spain in 2016 [16]. To date, only one published study performed in Tunisia reported a seroprevalence of 5% among slaughter workers considered to be an at-risk human population, suggesting silent circulation of CCHFv [15]. Thus, epidemiological surveys of ticks, humans, and domestic and wild animals for CCHFv in Tunisia are of major importance.

Recently, CCHFv has been detected in *Hyalomma aegyptium* collected from spur-thighed tortoises (*Testudo graeca*) captured in Algeria [23] and in Syria [39]. Thus, it is of particular epidemiological interest to study the impact of the high infestation intensity in *T. graeca* by *H. aegyptium* on the potential transmission of zoonotic pathogens such as CCHFv in the Mediterranean Basin. *Hyalomma aegyptium* has a three-host life cycle where larvae and nymphs infest a wide range of hosts including lizards, birds, small mammals [2, 25], and accidentally cattle [4], and humans [8, 42]. By contrast, adult *H. aegyptium* is host-specific to tortoises, particularly *T. graeca* [2, 22, 35, 37–40]. The geographical distribution of *H. aegyptium* covers the Mediterranean basin, the Balkans, and the Middle East [2, 25]; they are exotic elsewhere [7]. In addition to CCHFv, *H. aegyptium* is known to be a vector of *Hemolivia mauritanica* [38]. This tick species has been found to be infected with other human and animal pathogens such as *Anaplasma phagocytophilum*, *Ehrlichia canis*, *Coxiella burnetii* [31], and *Rickettsia aeschlimannii* [5].

Even though the spur-thighed tortoise is a protected species, it is commonly used illegally as a garden pet in Tunisia, in addition to being subject to thriving illegal trade between North Africa and Europe [6, 18]. The potential for *T. graeca* to transmit zoonotic pathogens is greater when *H. aegyptium* is infesting humans [8]. To increase our understanding of the epidemiology of CCHF, we assessed CCHFv infection in *H. aegyptium* collected from field-captured *T. graeca* in Tunisia.

## Materials and methods

### Ethics

The spur-thighed tortoise (*T. graeca*) is a protected species in Tunisia and subsequently any study involving this tortoise species must obtain prior approval from the General Directorate of Forest at the Tunisian Department of Agriculture. Our study was performed following approval from this authority (authorization no. 645, following an application submitted by Marwell Wildlife No. 1441 of 13 March 2017). Capture and handling of tortoises were performed by a trained veterinarian (C. Najjar) to minimize stress and pain.

### Tortoise search and tick collection

**Figure 1:**
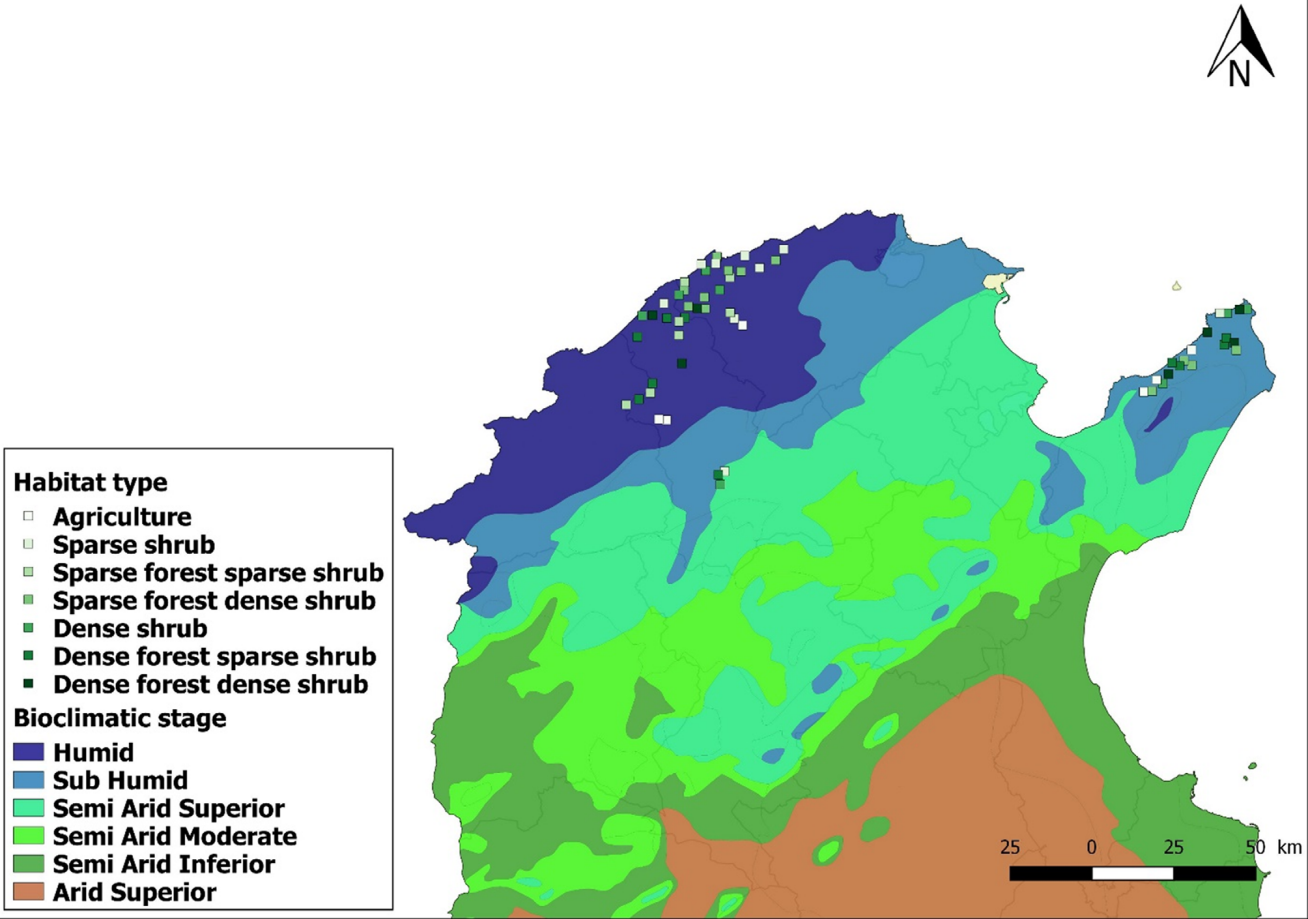
Map of the Tunisian bioclimatic stages and habitat types searched for free-ranging spur-thighed tortoises (squares).

Wild tortoises from different sites, located in humid and sub-humid bio-geographical areas of Northern Tunisia (Fig. 1), were sampled on foot transects conducted in their natural habitats between March and May 2017. Captured tortoises were identified on site, using identification keys [11, 19]: the endemic *T. graeca* has distinctive morphological traits that allow quick taxonomical identification. Tortoise age was also estimated by counting the scute rings (annuli) [9]. Each tortoise was examined thoroughly for the presence of ticks and released at the point of capture. The number of ticks and their attachment sites were registered for each tortoise. Collected ticks were placed in snap-cup vials and stored at −80° C until use. Tick species identification was performed on the whole tick load of a random sample of the captured tortoises, using the identification keys of Hoogstraal [20]; the remaining collected ticks were kept at −80° C to be examined later for the presence CCHFv. The number of tortoises screened was determined using Research Advisors’ sample size table corrected with the assumption that the proportion of *H. aegyptium* will be substantially different from 50% of the ticks collected, and allowing a subsequent reliable survey of pathogen infection incidence within the remaining ecto-parasites (Confidence Level = 95%; Margin of Error = 1%). Consequently, the ticks of 13.4% (*n* = 18) of the infested tortoises were identified, representing about 10% (*n* = 120) of the total collected.

### Molecular analysis

Ticks were examined for the presence of CCHFv by real-time reverse transcription (RT)-PCR [3]. Collected ticks were pooled according to the trapping location with a maximum of 10 individuals per pool for the non-engorged ticks and two per pool for engorged ticks, resulting in a total of 46 pools. The pools of ticks were transferred to PreCellys tubes containing silica beads and 0.5 mL of MEM and homogenized three times at 5600 rpm for 30 s, and finally centrifuged at 6000 rpm for 15 min. The total RNA was extracted with a Qiagen viral RNA mini kit from tick supernatant. The presence of CCHFv RNA was determined by qualitative real time RT-PCR performed on the S segment using the combination of the primer pairs CCHFV.S122F (5″CCT TTT TGA ACT CTT CAA ACC 3′)/CCHFV.S1R (5′TCT CAA AGA AAC ACG TGC C 3′) and the CCHFv probe (5′FAM 3′ACT CAA GGK AAC ACT GTG GGC GTA AG-BHQ1), as published previously [3]. The real time RT-PCR was performed using the SuperScript III Platinum one step Quantitative RT-PCR system (Invitrogen Life Technologies), according to the manufacturer’s instructions. One high and one low CCHFv viral RNA were used as positive controls. A volume of 5 μL of total extracted RNA was used for CCHFv genome detection.

## Results and discussion

A total of 147 tortoises (63 males, 72 females, 12 juveniles) were captured and identified as *T. graeca*. A total of 1174 ticks were collected. 10% of the collected ticks (*n* = 120) taken from 18 randomly selected tortoises were used for tick identifications: all were identified as adult *H. aegyptium*; there were 88 males and 32 females. Of a total of 147 examined tortoises, 134 were infested with at least one tick, yielding an infestation prevalence of 91.16%. The overall infestation intensity and abundance was 8.5 and 7.8, respectively. We observed higher overall infestation intensities compared to those reported from Algeria (1.7–9.4 [41]), Jordan (0.2–5.9 [32]), the Balkans (1.3 [37]), Russia (5.2 [33]), Italy (3.9 on tortoises imported from North Africa [6]), Tunisia (4.3 on tortoises seized by customs [18]), and Morocco (6.67 [35]). Our results provide strong evidence of the high dependence of adult *H. aegyptium* on its host *T. graeca*.

In the present study, no CCHFv was detected in *H. aegyptium*. In Tunisia, we previously reported the absence of CCHFv in *Hyalomma dromedarii* and *Hyalomma scupense* [15]. It is of key epidemiological importance to point out that reported rates of the virus infection in ticks from endemic areas varied from 0 to more than 50%. In Iran, the infection prevalence of field-collected *Hyalomma* sp. from Yazd province was 5.7% [45]. In Turkey, from a total of 250 *Hyalomma* sp. collected from humans with tick bites in an endemic focus of CCHF (25 pools with 10 ticks in each pool) and tested for CCHFv, seven pools were positive, yielding an infection rate of 28% [8]. In Spain, among 117 *Hyalomma lusitanicum* specimens collected from red deer, two pools were positive for CCHFv, yielding a minimum infection rate of 1.7% [13]. Following an outbreak of CCHF in Gujarat, India in 2011, an entomological investigation showed that the infection rate of *Hyalomma anatolicum anatolicum* with CCHFv was 1.4% [28]. From Kosovo, significantly different virus infection rates varying from 0 [14] to 15% [36] were reported.

While recent reports have demonstrated CCHFv detection in *H. aegyptium* removed from *T. graeca* captured in Algeria [23] and in Syria [39], in our study, real time RT-PCR analysis failed to detect the presence of CCHFv RNA, indicating that adult *H. aegyptium* were not carrying CCHFv. Based on the large number of tick and tortoise samples tested (>1000 ticks and >150 tortoises), our negative results strongly suggest the absence of CCHFv in *H. aegyptium*. Therefore, our findings are in contradiction with those from previous studies [23, 39]. Similar results were reported by another study performed in endemic foci for CCHF in Turkey, showing the absence of CCHFv in *H. aegyptium* collected from tortoises (*T. graeca*) [24]. Immature ticks collected from *T. graeca* captured in an endemic focus in Bulgaria were also negative for CCHFv [26]. Several species of *Hyalomma* collected from endemic foci located in Northern Turkey including *H. anatolicum*, *H. detritum*, and *H. marginatum* were infected with CCHFv, but not *H. aegyptium* collected from *T. graeca* [1]. Taking into account that the life cycle of *H. aegyptium* is not related to livestock but mainly to tortoises, this tick species is unlikely to play a major role in the epidemiology of CCHF.

In conclusion, the extension of CCHF to the Western Mediterranean Basin represents a serious public health threat. This extension is probably related to livestock trade and the spread of infected ticks by migratory birds [30]. Thus, prevention and control of CCHFv are needed to curb the extension of CCHF. *Hyalomma marginatum* is considered the main vector of CCHFv in the Western Palearctic region [12], and is located mainly in Northwestern Tunisia [27], an area that was not investigated during the study period. Therefore, screening of this tick species and its domestic animal hosts should continue in an effort to identify and confirm the presence of CCHFv circulating in Tunisia.
